# Esketamine Exposure Impairs Cardiac Development and Function in Zebrafish Larvae

**DOI:** 10.3390/toxics12060427

**Published:** 2024-06-13

**Authors:** Shuo Huang, Jingyi Wang, Tingting Lin, Chengyong He, Zhiyuan Chen

**Affiliations:** 1 The Second Affiliated Hospital of Fujian Medical University, Quanzhou 362000, China; huangshuoxm@163.com; 2 State Key Laboratory of Cellular Stress Biology, School of Life Sciences, Xiamen University, Xiamen 361102, China; 18965425636@163.com (J.W.); lin.0829@foxmail.com (T.L.)

**Keywords:** developmental toxicity, esketamine, zebrafish embryo, cardiac toxicity

## Abstract

Esketamine is a widely used intravenous general anesthetic. However, its safety, particularly its effects on the heart, is not fully understood. In this study, we investigated the effects of esketamine exposure on zebrafish embryonic heart development. Zebrafish embryos were exposed to esketamine at concentrations of 1, 10, and 100 mg/L from 48 h post-fertilization (hpf) to 72 hpf. We found that after exposure, zebrafish embryos had an increased hatching rate, decreased heart rate, stroke volume, and cardiac output. When we exposed transgenic zebrafish of the Tg(*cmlc2:EGFP*) strain to esketamine, we observed ventricular dilation and thickening of atrial walls in developing embryos. Additionally, we further discovered the abnormal expression of genes associated with cardiac development, including *nkx2.5*, *gata4*, *tbx5*, and *myh6*, calcium signaling pathways, namely *ryr2a*, *ryr2b*, *atp2a2a*, *atp2a2b*, *slc8a3*, *slc8a4a*, and *cacna1aa*, as well as an increase in acetylcholine concentration. In conclusion, our findings suggest that esketamine may impair zebrafish larvae’s cardiac development and function by affecting acetylcholine concentration, resulting in weakened cardiac neural regulation and subsequent effects on cardiac function. The insights garnered from this research advocate for a comprehensive safety assessment of esketamine in clinical applications.

## 1. Introduction

Ketamine is made up of two enantiomers that combine to form a racemic mixture: the dextrorotatory [S (+) ketamine] and the levorotatory [R (−) ketamine]. In many medical fields, such as anesthesia for pediatric and obstetric patients, acute and chronic pain patients management, and treatment of people with psychiatric disorders, esketamine has been widely used as a dextroisomer [1,2]. Ketamine, however, has been linked to serious adverse effects. Cardiotoxic effects are of great concern. Ketamine can not only produce a sympathomimetic effect through direct excitation of the sympathetic nerve center, stimulate the cardiovascular system, and produce a positive inotropic effect, but also directly inhibit cardiovascular function and produce a negative inotropic effect through direct inhibition of myocardium and vascular smooth muscle [3,4]. Moreover, early ketamine exposure in Xenopus embryos caused cardiac enlargement and heart dysfunction [5]. Examining esketamine’s cardiac effects is vital to ensure its safe use in light of these.

The common adverse reactions of esketamine in clinical are similar to those of ketamine, including dizziness, nausea, vomiting, cardiovascular excitation, visual impairment, and dissociation phenomenon, and are dose-dependent [6]. Cardiovascular system inhibition is also shown in severe and shock patients [7]. It has been shown that esketamine exposure during lactation and pregnancy in mammals impairs neuronal development in the progeny, leading to neuronal apoptosis and long-lasting cognitive deficits [8]. Furthermore, long-term esketamine exposure during pregnancy causes behavioral abnormalities, memory impairment, and a decrease in synaptic spine density in the offspring of pregnant rats [9,10]. Overall, the widespread use of esketamine and its potential toxicity have attracted people’s great attention. However, previous studies have mostly focused on the neurotoxicity of esketamine. Evaluation of esketamine cardiac developmental toxicity remains limited. Considering the widespread use of esketamine in children during the perioperative period, it is urgent to investigate the potential effects and mechanisms of esketamine on cardiac development.

The zebrafish is a popular choice for animal models studying human diseases because of its genetic similarity to humans [11], strong reproductive ability, quick growth, and transparent embryos that allow easy observation [12,13]. Heart activity in zebrafish starts at 22 hpf, but the heart valve function, which is closely related to heart function, is not developed until 48 hpf [14]. After that, the cardiovascular system in zebrafish reaches its maximum potential and displays complex metabolic pathways and ion channels [15].

Esketamine has an LD50 value of 2471.5 mg/L for zebrafish embryos at 96 hpf, and 548 mg/L is the maximum safe concentration for prolonged exposure to esketamine [16]. Adult zebrafish exhibit anxiety-like behaviors when exposed to 20 mg/L of ketamine [17]. Here, we use the zebrafish model to investigate the effects and underlying mechanisms of esketamine at 1, 10, and 100 mg/L on cardiac development from 48 hpf to 72 hpf. The findings suggest that zebrafish embryos exposed to eketamine have some degree of cardiac developmental toxicity. This work aims to examine the toxic mechanisms of eketamine and how it affects the cardiac development of zebrafish embryos. Improving knowledge about this medicine is essential to offer strong backing for responsible drug use.

## 2. Materials and Methods

### 2.1. Chemicals

Esketamine Hydrochloride Injection, with a high-performance liquid chromatography (HPLC) purity of ≥98%, was purchased from Hengrui Pharmaceutical Co., Ltd. (Lianyungang, Jiangsu, China) (2 mL:50 mg, Batch No. H20193336). The remaining chemicals used in the investigation were all analytical grade and came from outside vendors.

### 2.2. Zebrafish Culturing and Exposure

We strictly followed the requirements of the Animal Ethics Committee of Xiamen University and adhered to the standard procedures in our laboratory for the maintenance of wild-type TU zebrafish. The fish were kept under the following conditions: a 14-h light/10-h dark cycle, water temperature of (28 ± 1) °C, pH range of 7.0–7.4, and dissolved oxygen level of 7–8 mg/L. Wild-type zebrafish are kept until they reach adulthood (approximately 2.5–4 months after fertilization), with males and females kept separately. After reaching sexual maturity, healthy and high-quality spawning individuals were selected for egg collection. In the evening, male and female zebrafish were separated by a divider (with a ratio of 2 females to 1 male), and the divider was removed the next morning after 9 am to allow free mating and egg laying. Embryos that were newly laid, between 0.5 and 1.0 hpf, were used for embryo exposure experiments.

### 2.3. Zebrafish Embryo Exposure

The freshly prepared pre-oxygenated (dissolved oxygen concentration of 7–8 mg/L) The common E3 medium (containing 3.5 g/L NaCl, 0.05 g/L NaHCO_3_, 0.05 g/L KCl, and 0.05 g/L CaCl_2_) was used to dilute the stock solution of esketamine, resulting in exposure solutions with concentrations of 1, 10, and 100 mg/L. Embryos at 0.5–1.0 hpf were transferred to glass culture dishes with a diameter of 90 mm containing 20 mL of culture solution. Each dish contained 30 embryos and 4 petri dishes per concentration group (*n* = 4). After 48 h, the culture medium was replaced with the esketamine exposure solutions of different concentrations.

### 2.4. Developmental Toxicity in Zebrafish

At 60 and 72 hpf, the rates of hatching and mortality were evaluated. The calculation method is based on previous studies, and the following formula was adopted [18]: Mortality rate (%) = (deathed embryo number/total embryo number) × 100%. Hatching rate (%) = (hatched embryo number/total embryo number) × 100%

We placed the larvae in the SMZ168 stereozoom microscope field and recorded the heartbeats automatically using DanioScope 1.1 (Noldus IT, Wageningen, The Netherlands) for 20 s each, then converted them to the number of larvae’s heart rate (HR) per minute. At the same time, images were extracted from these visual frequencies to measure the long (a) and short (b) axis lengths of the diastolic and systolic ventricles. And then calculate end-diastolic volume (EDV), end-systolic volume (ESV), stroke volume (SV), and cardiac output (CO) of the larvae. The common formula for EDV and ESV is: volume = 4/3π ab^2. The calculation formula for SV is: SV = EDV − ESV, and the calculation formula for CO is: CO = SV × HR.

### 2.5. Transgenic Zebrafish Imaging

The transgenic Tg(*cmlc2:EGFP*) zebrafish expresses enhanced green fluorescent protein (EGFP) specifically in cardiac muscle cells, providing the possibility for detailed observation of cardiac morphology in larvae. These fish were kindly provided by Professor Bo Zhang from Peking University. At 72 hpf, 10 larvae were sampled for each group; it was placed in the field of view of the Zeiss LSM 900+ Airyscan2 confocal microscope (Oberkochen, Germany), which was used to capture images of the zebrafish heart.

### 2.6. Real-Time Quantitative PCR (RT-qPCR)

At 72 hpf, 15 larvae were pooled as a subsample for each group, following our previously reported method for mRNA expression analysis using qPCR [19,20]. We chose β-actin as the reference gene because, as one of the butler genes, it is expressed in all cells and tissues, and its level is constant, so it is more accurate to use it as a reference. Total RNA was extracted using TRIzol reagent (Takara, Tokyo, Japan) according to the manufacturer’s instructions. The first-strand cDNA was synthesized using the SuperMix kit (TransGen, Beijing, China). qPCR analysis was performed using TransStart Tip Green qPCR SuperMix (Transgen, Beijing, China) following the manufacturer’s protocol. The expression levels of *nkx2.5*, *gata4*, *tbx5*, and *myh6*, *ryr2a*, *ryr2b*, *atp2a2a*, *atp2a2b*, *slc8a3*, *slc8a4a*, and *cacna1aa*, *grin1a*, *grin1b*, *grin2ca*, and *grin2bb* genes were calculated using the 2^−ΔΔCt^ method. The primer sequences used are listed in Supplementary Materials, Table S1.

### 2.7. The Level of Ach and Activities of AchE and ChAT

A hundred larvae per Petri dish per concentration group were collected at 72 hpf to measure the level of acetylcholine (Ach), was purchased from Jiancheng Biology Engineering Institute (Nanjing, Jiangsu, China) (48T, Art.No. A105-1-1), activities of acetylcholinesterase (AchE), was purchased from Jiancheng Biology Engineering Institute (Nanjing, Jiangsu, China) (50 tubes/24 samples, Art.No. A024-1-1) and choline acetyltransferase (ChAT), was purchased from Jiancheng Biology Engineering Institute (Nanjing, Jiangsu, China) (40 tubes/20 samples, Art.No. A079-1-1). The specific testing procedures followed the BCA Protein Quantification Assay Kit Manual guidelines from Yaenzyme Bioengineering Co., Ltd. and the Ach, AchE, and ChAT Assay Kit Manual from Jiancheng Bioengy Engineering Institute Co., Ltd. (Nanjing, Jiangsu, China).

### 2.8. Statistical Methods

Statistical analysis was executed by applying Graphpad Prism 7.0 (GraphPad Software, Boston, MA, USA). All data were checked for normality by *Kolmogorov–Smirnov* test and homogeneity by *Levene’s* test. Data are shown as the means ± standard error of the means (SEM).

One-way analysis of variance (ANOVA) was performed, followed by *Tukey’s* multiple comparison test to assess significant differences (*p* < 0.05) between the exposure groups and the control group. To analyze the differences in hatching rates and mortality after embryo exposure, the time and concentration were used as variables. Two-way ANOVA was used to analyze death and hatching rates, followed by *Tukey’s* multiple comparison test.

## 3. Results

### 3.1. Esketamine Affected the Development of Zebrafish

**Figure 1:**
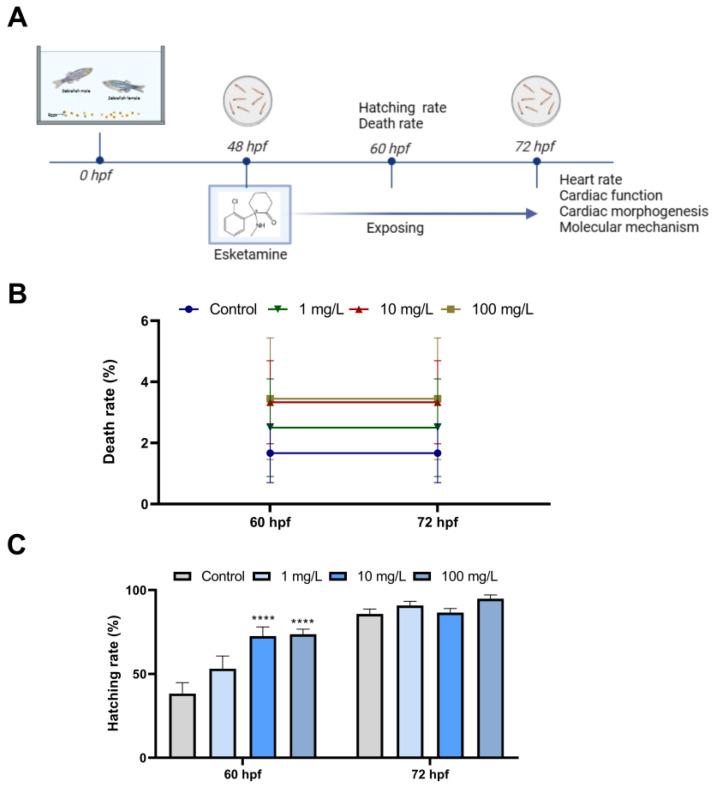
Developmental effects of zebrafish embryos exposure to esketamine. (**A**) Scheme of experiment design. (**B**) Mortality rate from 60 to 72 hpf. (**C**) Hatching rate from 60 to 72 hpf A two-way analysis of variance (ANOVA) was employed for the statistical analysis of the death and hatching rates. This was followed by multiple comparison tests, and the results were expressed as the mean ± SEM (*n* = 4). **** *p* < 0.0001.

We examined the mortality and hatching rates of zebrafish larvae at 60 hpf and 72 hpf after esketamine exposure for the evaluation of possible effects of esketamine toxicity on zebrafish embryonic development. As shown in Figure 1B, there was no discernible mortality compared with the control group. The hatching rate of zebrafish larvae exposed to 10 mg/L and 100 mg/L esketamine at 60 hpf were all marginally higher than that of the control group (Figure 1C).

### 3.2. Esketamine Impaired the Heart Function of Zebrafish

**Figure 2:**
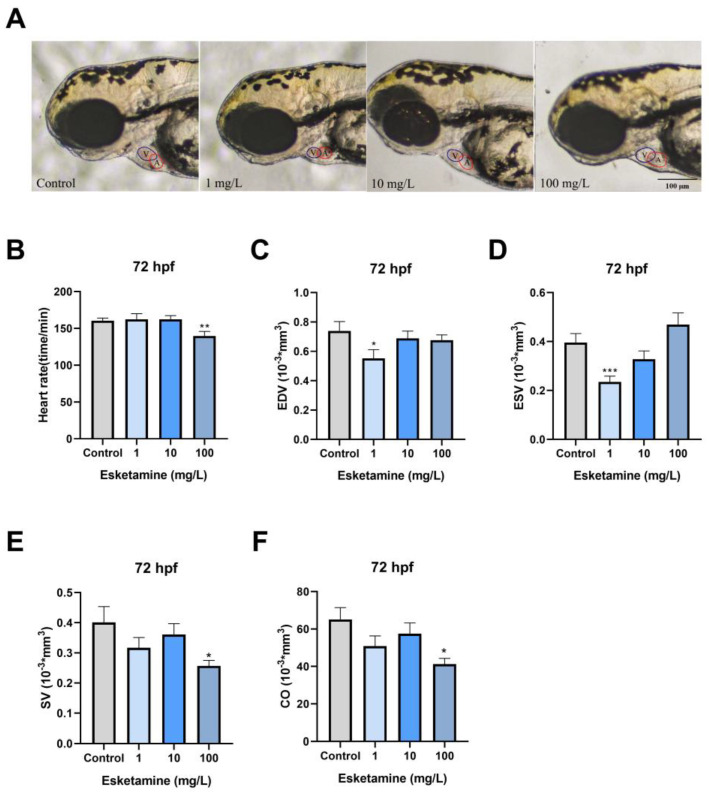
Cardiac function of zebrafish larvae exposed to esketamine at 72 hpf. (**A**) Morphological alterations were observed using microscopy (Scale bar: 100 µm), (**B**) heart rate, (**C**) end-diastolic volume (EDV), (**D**) end-systolic volume (ESV), (**E**) stroke volume (SV), and (**F**) cardiac output (CO). The data underwent a one-way analysis of variance (ANOVA) and were further evaluated using Tukey’s multiple comparisons test. Results are reported as the mean ± SEM (*n* ≥ 25). * *p* < 0.05, ** *p* < 0.01, *** *p* < 0.001.

First, we looked for morphological changes in the heart, like cardiac tubification, atrial stenosis, and pericardial edema. There were no discernible significant morphological alterations, as shown in Figure 2A. The zebrafish’s heart rate was then recorded at 72 hpf. The cardiac rhythm in the 100 mg/L esketamine groups showed a significant decrease (Figure 2B). We then assessed parameters like EDV, ESV, SV, and CO that are suggestive of cardiac blood circulation function. The 1 mg/L exposure group significantly reduced EDV and ESV (Figure 2C,D). ESV values for the control group were 0.39 ± 0.04 mm^3^, while the 1 mg/L exposure group’s values were 0.23 ± 0.02 mm^3^. In the group exposed to 100 mg/L, both SV and CO demonstrated a remarkable decrease (Figure 2E,F). SV was 0.40 ± 0.05 mm^3^ in the comparison group and substantially decreased to 0.25 ± 0.02 mm^3^ in the 100 mg/L exposed groups; CO in the 100 mg/L exposed group was also significantly lower at 41.22 ± 3.05 mm^3^ compared to 65.06 ± 6.34 mm^3^ in the control group. Ultimately, the information points to a significant impairment of zebrafish larvae’s cardiac function caused by esketamine.

### 3.3. Esketamine Altered the Cardiac Morphogenesis of Zebrafish

**Figure 3:**
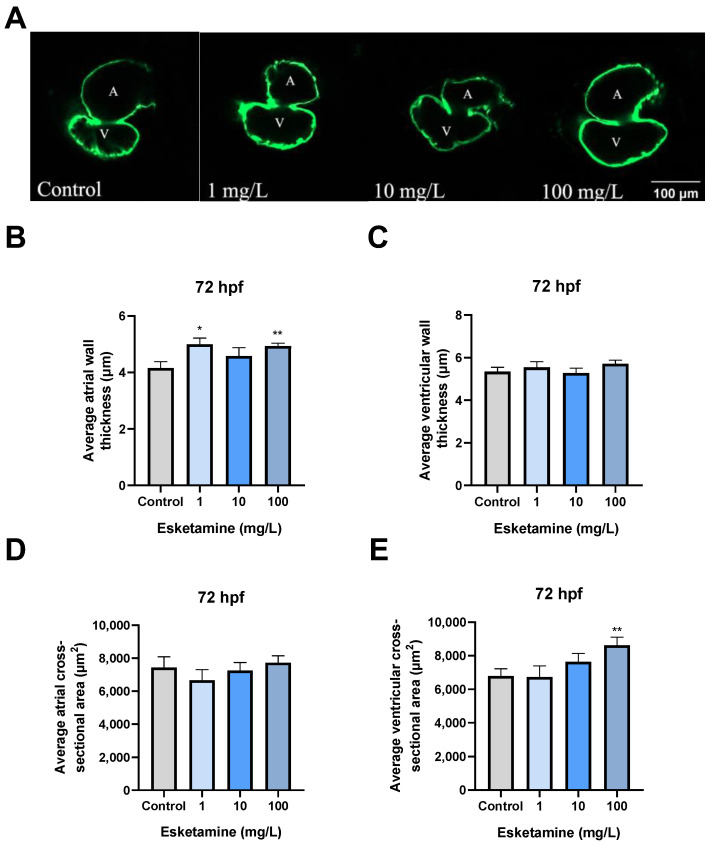
Cardiac morphogenesis of zebrafish larvae exposed to esketamine at 72 hpf. (**A**) Heart structure visuals showcase the cardiac morphogenesis in larvae exposed to esketamine. The measurements include (**B**) the average thickness of the atrial wall, (**C**) the average thickness of the ventricular wall, (**D**) the average cross-sectional area of the atrium (noted as A for atrium and V for ventricle), and (**E**) the mean cross-sectional area of the ventricles. Following the statistical analysis with a one-way analysis of variance (ANOVA) and a Tukey multiple comparisons test, the results are reported as the mean ± SEM (*n* = 10). * *p* < 0.05, ** *p* < 0.01.

We employed Tg(*cmlc2:EGFP*) zebrafish to investigate the specific alterations in cardiac morphology resulting from exposure to esketamine. When young zebrafish were exposed to 1, 10, and 100 mg/L esketamine, their atrial wall thickness increased significantly from 4150.75 ± 0.23 µm^2^ to 4992.96 ± 0.23, 4582.43 ± 0.29, and 4931.43 ± 0.11 µm^2^, respectively, in comparison to the control group (Figure 3A,B). Furthermore, exposure to 100 mg/L esketamine resulted in a significant increase in the ventricular cross-sectional area, from 6783.91 ± 436.4 µm^2^ to 8630.31 ± 470.2 µm^2^, in comparison to the control group (Figure 3A,E). All of these results point to the possibility that esketamine may have teratogenic effects on the development of the heart in zebrafish larvae.

### 3.4. Esketamine Exposure Modified the Expression of Genes Linked to Heart Development

**Figure 4:**
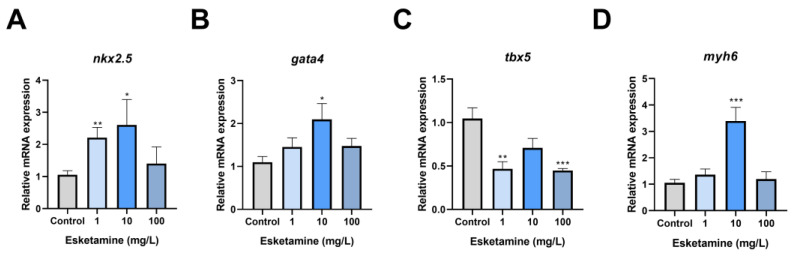
The expression levels of cardiac development-related genes in zebrafish larvae exposed to esketamine at 72 hpf. (**A**) *nkx2.5*, (**B**) *gata4*, (**C**) *tbx5*, and (**D**) *myh6*. The data underwent a one-way analysis of variance (ANOVA) and a Tukey multiple comparisons test. The findings are expressed as the mean ± SEM *(n* = 3). * *p* < 0.05, ** *p* < 0.01, *** *p* < 0.001.

We evaluated the manifestation of critical genes for cardiac development in zebrafish larvae exposed to esketamine for 72 hpf to uncover the molecular pathways behind esketamine-induced cardiac abnormalities. The relative expression levels of *gata4*, *myh6*, and *nkx2.5* showed an upward trend and a similar pattern in the group exposed to esketamine. In particular, *nkx2.5* transcription levels in the 1 mg/L esketamine group showed 2.09-fold higher than in the control group, whereas the 10 mg/L group showed a significant rise to 3.01-fold (Figure 4A). Although the levels in this group decreased slightly compared to the control group, the difference was not statistically significant. While the 1 mg/L and 100 mg/L groups showed an increasing trend but did not reach statistical significance, the *gata4* transcription levels peaked in the 10 mg/L groups, showing a 1.91-fold increase over the control group (Figure 4B). On the other hand, the 100 mg/L groups showed a significant reduction in *tbx5* expression, with a relative expression level of just 0.448, compared to 0.42 times in the control group (Figure 4C). The *myh6* expression levels rose dramatically to 2.31 times those of the control group in the 10 mg/L. However, in the 1 mg/L and 100 mg/L groups, they fell to 1.29 and 1.13 times those of the control group individually (Figure 4D). In conclusion, the results point to the abnormal expression of genes involved in cardiac development as the cause of esketamine’s effects on cardiac development.

### 3.5. Esketamine Exposure Decreased the Expression of Genes Associated with Calcium Signaling Pathway

**Figure 5:**
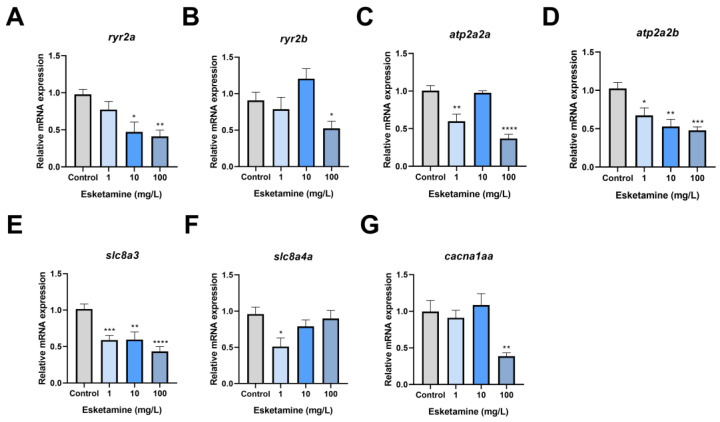
The expression levels of the calcium signaling pathway genes in zebrafish larvae exposed to esketamine at 72 hpf. (**A**) *ryr2a*, (**B**) *ryr2b*, (**C**) *atp2a2a*, (**D**) *atp2a2b*, (**E**) *slc8a3*, (**F**) *slc8a4a*, and (**G**) *cacna1aa*. The expression values of these genes were normalized using *gapdh*. The evaluation of the data was carried out using Tukey’s multiple comparisons test, and the findings are represented as the mean ± SEM (*n* = 3). * *p* < 0.05, ** *p* < 0.01, *** *p* < 0.001 and **** *p* < 0.0001.

When esketamine was administered to zebrafish hearts, SV and CO decreased in the context of the observed cardiac developmental toxicity. As a result, we evaluated the central markers’ gene expression in the calcium signaling pathway. According to our research, there was a dose-dependent decrease in the relative transcription level of *ryr2a* after exposure to esketamine. In the group exposed to 100 mg/L, this reduction was particularly noticeable, reaching 42% of the control level (Figure 5A). However, after exposure, there appeared to be a disruption in the relative expression level of *ryr2b* (Figure 5B). Following exposure, *atp2a2a*’s relative expression level was stable at 10 mg/L but dramatically dropped to 36% and 59% of the control level at 1 mg/L and 100 mg/L, respectively (Figure 5C). Furthermore, *atp2a2b* expression level showed a dose-dependent decline, with the group exposed to 100 mg/L experiencing a significant decrease to 46% of the control level (Figure 5D). Moreover, Figure 5E shows a decline in *slc8a3* expression, with levels of 57%, 58%, and 42% of the control level, respectively. The *slc8a4a* expression also dramatically dropped to 53% of the control level in the 1 mg/L group (Figure 5F). Moreover, the 100 mg/L group’s relative transcription level of *cacna1aa* was 38% of the control level, which was significantly lower in the exposed group (Figure 5G). These results suggest that esketamine exposure modifies the expression of genes linked to calcium channels in zebrafish larvae, significantly impacting calcium homeostasis. 

### 3.6. Esketamine Exposure Did Not Affect the Expression of Genes Linked to N-Methyl-D-Aspartate-Receptor (NMDAR)

**Figure 6:**
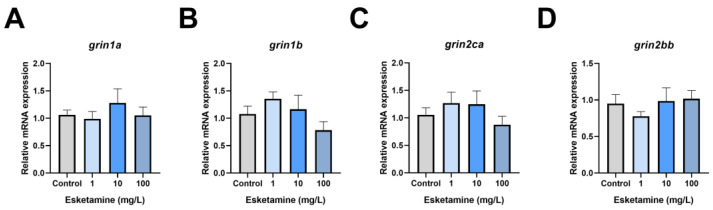
The expression levels of NMDAR-related genes in zebrafish larvae exposed to esketamine at 72 hpf. (**A**) *grin1a*, (**B**) *grin1b*, (**C**) *grin2ca*, and (**D**) *grin2bb*. The data underwent a one-way analysis of variance (ANOVA) and a Tukey multiple comparisons test. The findings are expressed as the mean ± SEM (*n* = 3).

Considering the primary target of esketamine action, we analyzed the expression of NMDAR-related genes in zebrafish larvae exposed to esketamine for 72 hpf. We found that esketamine exposure did not significantly change the mRNA levels of NMDAR-related genes in juvenile fish (Figure 6).

### 3.7. Exposure to Esketamine Raised Ach Concentrations and Changed the Activities of AchE and ChAT

**Figure 7:**
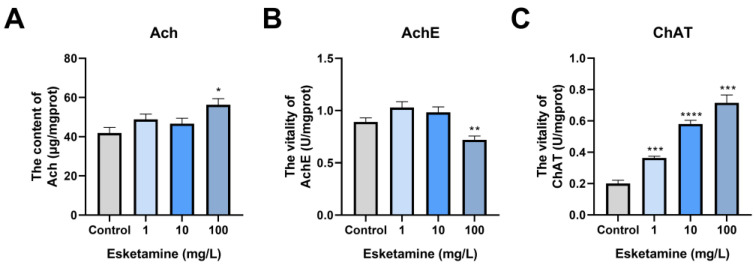
The level of Ach and activities of AchE and ChAT in zebrafish larvae exposed to esketamine at 72 hpf. (**A**) Ach. (**B**) AchE. (**C**) ChAT. The analysis of the data was performed using Tukey’s multiple comparisons test, and the results were expressed as the mean ± SEM *(n* = 3). * *p* < 0.05, ** *p* < 0.01, *** *p* < 0.001 and **** *p* < 0.0001.

Considering the inhibitory effects of esketamine exposure on cardiac function in zebrafish, we further investigated its neural regulatory mechanisms on the heart. Zebrafish larvae exposed to esketamine showed a trend toward higher Ach concentrations; levels rose to 1.16, 1.11, and 1.34 times that of the control group (Figure 7A). In the 1 mg/L and 10 mg/L groups, AchE activity showed an increasing trend; however, in the 100 mg/L groups, it significantly reduced, reaching 0.81 times the control level (Figure 7B). Following exposure, there was a dose-dependent increase in ChAT activity, with concentrations rising to 1.81, 2.90, and 3.57 times that of the control (Figure 7C). To sum up, the information indicates that zebrafish larvae’s exposure to esketamine modifies their Ach levels, which in turn impacts their neural regulatory systems.

## 4. Discussion

Cardiovascular effects are one of the main adverse reactions associated with the clinical use of esketamine. However, there are currently no reports on the cardiac safety of esketamine. Our research shows that esketamine exposure can cause abnormalities in the heart development of zebrafish embryos and that esketamine may affect neurohumoral regulation, affecting cardiac function.

Compound toxicity can be determined by zebrafish physiological markers at different developmental stages [21]. Evaluating hatching rates and mortality in zebrafish is common practice to determine developmental toxicity. Zebrafish embryo hatching rate correlates with spontaneous movement and hatching enzyme activity [22]. Previous studies have demonstrated that zebrafish exposed to plant toxins may experience higher mortality and lower hatching rates. Zebrafish embryo and larval death rates increased dose-dependent when exposed to ketamine at concentrations of 0.2, 0.4, and 0.8 mg/mL, according to FÉLIX et al.’s observations [23]. Our investigation found no discernible variation in the zebrafish embryo death rate between the esketamine exposed and control groups. This lack of variation is probably due to the low, safe esketamine concentration. But, after exposure to esketamine, we saw a marked increase in larval hatching at 60 hpf. This discovery is consistent with the findings published by Wenjuan Yuan et al. [16]. It is assumed that the increased hatching may be associated with esketamine-induced changes in enzyme activity and abnormal muscle responses [24,25].

Clinical studies have suggested that esketamine generally causes an increase in heart rate due to its central sympathomimetic activities [26]. However, other studies have observed that ketamine exposure may induce a decrease in heart rate within zebrafish larvae [27]. The current investigation found that 72 hpf zebrafish larvae exposed to esketamine showed impaired cardiac functionality. Specifically, after exposure to esketamine, there was no significant difference in the effect of 1.10 mg/L zebrafish embryo groups on heart rate, while a significant decrease was observed in the 100 mg/L group. The observed differences can be ascribed to the varying cardiovascular characteristics of embryos and adults, as well as to the impact of drug dosage on cardiovascular responses [28]. The significant decrease in SV and CO seen in the 100 mg/L concentration group may be explained by esketamine’s primary direct inhibitory effect on the heart at high doses, which is probably caused by inhibited calcium ion transport, sympathetic nerve blockade, or depletion of presynaptic catecholamine stores [29]. Furthermore, the effects of esketmine exposure on zebrafish cardiac morphogenesis were investigated using transgenic zebrafish. In zebrafish exposed to esketamine, our research showed a thickening of the atrial wall and dilation of the ventricles. Thus, we speculate that exposure to esketamine may reduce myocardial contractile function, which could impair cardiac function as represented by a decrease in SV and CO. To support this theory, we measured the amounts of Ach, the activities of AchE, the enzyme that breaks down Ach, and ChAT. We found that Ach levels had increased. In addition, we also detected the expression level of related genes of NMDAR, which is the target receptor of esketamine and found no significant changes. These supported our theory even more.

The development of zebrafish embryonic heart is regulated by several related genes. Among them, *nkx2.5*, *gata4*, and *tbx5* are three important cardiac transcription factors that play a crucial role in heart morphogenesis. Additionally, they also influence the development of the conduction system [30,31,32]. *nkx2.5* is expressed early among cardiac progenitor cells and is involved in every stage of heart development [33]. Upregulating cardiac-specific genes and enhancing cardiomyocytes’ contractile and electrophysiological properties, overexpression of *nkx2.5* significantly promotes the differentiation of human embryonic stem cells into the cardiac lineage [34]. *gata4* is primarily active during the early stages of heart and organ development, playing a pivotal role in heart formation, cardiomyocyte differentiation, and functional maintenance. Numerous investigations into the connection between *gata4* expression and heart development have shown that overexpression of *gata4* dramatically increases the differentiation of embryonic stem cells into cardiomyocytes and upregulates the expression of genes specific to the heart. Moreover, *gata4* overexpression increases the number of cardiomyocytes and encourages their growth [35]. *tbx5* is essential for the differentiation of cardiac contractile cells [32], and both overexpression and underexpression may result in cardiac malformations [36]. Our investigation found that *tbx5* was significantly downregulated, and *nkx2.5* and *gata4* were significantly upregulated. The heart abnormalities in young zebrafish exposed to esketamine are probably caused by this gene dysregulation. Previous studies have shown that transcription factors such as *tbx5* and *gata4* can regulate the expression of *myh6* [37]. In our study, we suggested that atrial dilation in zebrafish larvae following exposure to amisulpride may be associated with aberrant regulation of *myh6* expression.

Interestingly, in comparison to the control group, our study showed a significant increase in the expression levels of *gata4*, *myh6*, and *nkx2.5* in the 10 mg/L esketamine exposure group. However, in the 1 mg/L and 100 mg/L exposure groups, the increase was not significant. At the same time, the decreased expression level of *tbx5* also showed this performance. Hormesis, a dose-response phenomenon characterized by a low-dose stimulation in opposition to a high-dose inhibition and involves the activation of cellular defense mechanisms, may be responsible for this dose-dependent shift in the gene expression profile [38]. The stimulatory effects of common pesticides have also been reported in previous toxicological studies [39]. In our investigation, *nkx2.5*, *gata4*, and *myh6* expression were increased at lower esketamine concentrations, suggesting an initial stimulatory effect. On the other hand, increased esketamine concentrations might disrupt cellular signaling pathways or trigger compensatory mechanisms, ultimately resulting in the observed downregulation of genes.

**Figure 8:**
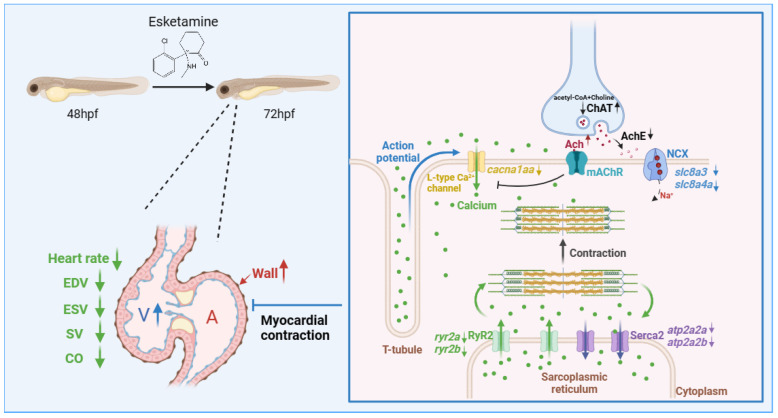
The proposed mechanism of esketamine affects the contraction of cardiomyocytes in zebrafish. Initially, esketamine exposure increased the concentration of Ach, which inhibits calcium channels, resulting in a decrease in the mRNA expression of *cacna1aa*, which are L-type voltage-gated calcium channels, leading to a decrease in the influx of extracellular calcium ions into the cytoplasm. As a result, the activation of the calcium channel RyR2 is delayed, requiring the attachment of calcium ions to the membrane of the sarcoplasmic reticulum (SR). Additionally, the mRNA expression of *ryr2a* and *ryr2b* also decreased after exposure. These combined effects severely impede the release of calcium from the SR in cardiac myocytes, resulting in a relative decrease in cytoplasmic calcium concentration. This relative decrease in calcium concentration disrupts the binding of calcium to myosin, limiting the sliding of myofilaments and ultimately impairing the contractile ability of cardiac myocytes.

Previous studies suggest that the suppression of intracellular Ca^2+^ release brought on by ketamine and the suppression of L-type voltage-gated Ca^2+^ channels, which lower intracellular Ca^2+^ concentrations, are responsible for the partial cardiac suppression brought on by esketamine [6]. Moreover, it has been shown that N-acetylcysteine protects zebrafish embryonic 5-HT neurons from the cytotoxic effects of ketamine; however, this neuroprotection may depend on intracellular calcium concentrations [27]. Based on these results, we then verified the gene abnormal expression linked to calcium signaling in zebrafish after esketamine exposure to assess the mechanisms of esketamine-induced cardiac toxicity in larvae. We propose that esketamine impairs cardiac function by acting on the calcium signaling pathway (as illustrated in Figure 8).

An essential neurotransmitter in the control of heart rate is Ach. It attaches itself to myocardial cell membrane M2 receptors. On the one hand, it causes an efflux of K^+^, which lowers the heart rate and reduces the sinus node’s automaticity. On the other hand, it directly blocks calcium (Ca^2+^) channels, lowering calcium influx and weakening myocardial contraction even more [40]. In our investigation, we found that exposure to esketamine significantly raised the concentration of Ach. This is probably caused by a significant rise in ChAT concentration and a corresponding fall in AchE concentration. This clarifies the decrease in heart rate brought on by esketamine exposure, as well as the reduced expression of the calcium channel *ryr2*, which is dependent on the association with calcium ions on the sarcoplasmic reticulum (SR) membrane [41], and *cacna1aa*, which is connected to L-type voltage-gated calcium channels [42]. On the other hand, Na^+^/Ca^2+^ exchanger (NCX) functions as the main pathway for calcium ion extrusion during muscle relaxation, while sarcoplasmic reticulum Ca^2+^-ATPase 2a (Serca2a) promotes the translocation of calcium ions from the cytoplasm into the sarcoplasmic reticulum [43], maintaining an ideal intracellular calcium ion concentration [44]. Here, the reduced expression of *slc8a3*, *slc8a4a*, *atp2a2a*, and *atp2b2b* may prevent calcium from leaving the cytoplasmic matrix quickly. This might be an adaptive mechanism to maintain cellular homeostasis after the calcium release is disrupted. According to our research, esketamine might impact calcium signaling by modifying acetylcholine levels, which might then impact juvenile zebrafish cardiac function.

Our study does have certain limitations, though. Our observations and analytical results from the experiments are the only foundation for these interpretations. More research is needed to understand how esketamine affects zebrafish heart function.

## 5. Conclusions

Overall, this study shows that esketamine has significant cardiac developmental toxicity in zebrafish. On one hand, esketamine affects the cardiac structure, possibly by altering the expression of genes related to cardiac development. On the other hand, esketamine increases the Ach level and impairs the calcium signaling pathway, both of which decrease heart function. By deepening our knowledge of esketamine’s toxic effects and mechanisms, this research may contribute to the development of safe strategies for clinical drug use. Moreover, these results provide insightful information for protecting public health.

## Data Availability

Data will be made available on request.

## Supplementary Materials

The following supporting information can be downloaded at: https://www.mdpi.com/article/10.3390/toxics12060427/s1. Table S1: List of primers used for qPCR.
