# Supplementary material for: Esketamine Exposure Impairs Cardiac Development and Function in Zebrafish Larvae
Source: Toxics. 2024 Jun 13;12(6):427. doi: 10.3390/toxics12060427 (PMC11209413; doi:10.3390/toxics12060427)
Supplement: Supplementary file 1 [file toxics-12-00427-s001.zip › toxics-3042942 supplementary.pdf]

Table S1

Sequence of primers used for reverse transcription-quantitative PCR.

Gene Name	Forward primer 5′- 3′	Reverse primer 5′- 3′	Genbank number
gapdh	GACGCGGGTGCTGGTATTGCT	CTACTCCTTGGAGGCCATGTGT	NM_002046.7
gata4	CAGGCGGGTGGGTTTAT	TGGTTCAGTCTTGATGGGTC	DQ886664
tbx5	ATTCGCCGATAACAAATGG	CGCCTTGACGATGTGGAT	AF152607
nkx2.5	GTCCAGGCAACTCGAACTACTC	AACATCCCAGCCAAACCATA	NM131421
myh6	TGATTTCCCAACTTACCCG	CCATGTTGCCACCTCTGTA	NM_198823.1
cacna1aa	GGTGGCCGCAAGTGCTAAAACA	TGAAGTTGTTCCCAGCTCTGTGAC	XM_021474441.1
ryr2a	AGGACTCAAGCCAAATCGAG	TCACGACCATGTCCTTCTGA	XM_009300703
ryr2b	TCCTTTAGTTTTTTAAGCGAGA	GTCCATCGAACTCCAGTTTACG	XM_017351708
atp2a2a	ATTTACTTGTGCGATTCTTCTAC	CACGATGTCTTTGGCTTTGA	BC_045327
atp2a2b	GAAATAAAGTCCCAATGACGC	ACATGCCAACACAAGCCCAC	NM_001030277.1
slc8a3	GAGGAAGCAAGAAGAATAGC	AGTCAAAACAAGATGGCAGA	XM_005156999.4
slc8a4a	GGAAGAGACGGAGAAGAGC	ATGGTGAAGAGGGTGACGGA	NM_001089419.1
